# Milk Protein-Derived Antioxidant Tetrapeptides as Potential Hypopigmenting Agents

**DOI:** 10.3390/antiox9111106

**Published:** 2020-11-10

**Authors:** Saerom Kong, Hye-Ryung Choi, Yoon-Jeong Kim, Yoon-Sik Lee, Kyoung-Chan Park, Seon-Yeong Kwak

**Affiliations:** 1 School of Chemical and Biological Engineering, Seoul National University, Seoul 08826, Korea; cherry0608@snu.ac.kr; 2 Department of Dermatology, Seoul National University Bundang Hospital, Seongnam 13620, Korea; hyeryung.choi@gmail.com (H.-R.C.); kyj5681@welskin.co.kr (Y.-J.K.); 3 Medical Science Research Institute, Seoul National University Bundang Hospital, Seongnam 13605, Korea; 4 Department of Dermatology, College of Medicine, Seoul National University, Seoul 03080, Korea; 5 Department of Agriculture, Forestry and Bioresources, College of Agriculture and Life Science, Seoul National University, Seoul 08826, Korea; 6 Research Institute of Agriculture and Life Sciences, Seoul National University, Seoul 08826, Korea

**Keywords:** milk-protein derived tetrapeptides, hypopigmenting, tyrosinase inhibitor, antioxidant

## Abstract

Excessive accumulation of melanin can cause skin pigmentation disorders, which may be accompanied by significant psychological stress. Although many natural and synthetic products have been developed for the regulation of melanogenesis biochemistry, the management of unwanted skin pigmentation remains challenging. Herein, we investigated the potential hypopigmenting properties of peptide sequences that originated from milk proteins such as ĸ-casein and β-lactoglobulin. These proteins are known to inhibit melanogenesis and their hydrolysates are reported as antioxidant peptides. We synthesize tetrapeptide fragments of the milk protein hydrolysates and investigate the amino acids that are essential for designing peptides with tyrosinase inhibitory and antioxidant activities. We found that the peptide methionine-histidine-isoleucine-arginine amide sufficiently inhibits mushroom tyrosinase activity, shows potent antioxidant activity and effectively impedes melanogenesis in cultured melanocytes via cooperative biological activities. Our findings demonstrate the potential utility of the bioactive tetrapeptide from milk proteins as a chemical alternative to hypopigmenting agents.

## 1. Introduction

Melanin is a pigment molecule that is synthesized by melanocytes in the basal layer of the epidermis, which displays coloring and plays a vital role in protecting skin from UV light radiation and in scavenging toxic chemicals [1,2,3]. However, overproduction and excessive accumulation of melanin can cause skin pigmentation disorders such as melasma, freckles, age spots and solar lentigo, which may be accompanied by significant psychological stress. Thus, substantial efforts have been devoted to the identification of factors that regulate melanogenesis and the development of active hypopigmenting agents. Melanogenesis is a complex biochemical reaction that is affected by extrinsic and intrinsic factors such as hormonal changes, inflammation, age and exposure to UV light. The most common hypopigmentation strategy is tyrosinase inhibition since melanin synthesis begins with the oxidation of the amino acid L-tyrosine to L-3,4-dihydroxyphenylalanine (DOPA), which is catalyzed by tyrosinase and is a rate-determining step for the production of melanin. The tyrosinase activity is typically decreased either by chelating dinuclear copper at the enzyme active site using direct tyrosinase inhibitors or by reducing DOPA-quinone products using antioxidants. Although human tyrosinase is different from mushroom tyrosinase [4], mushroom tyrosinase has been used extensively as a model system for screening tyrosinase inhibitors due to its similarity to human tyrosinase, well-characterized structure and biochemical properties [5]. Another primary strategy is to impede the binding of α-melanocyte stimulating hormone (α-MSH) to the melanocortin 1 receptor (MC1R), which can induce tyrosinase expression by activating the signaling pathway. Agouti-signaling protein is reported to block the binding of α-MSH and α-MSH analogs could be used to regulate melanogenesis activity [6,7,8].

A variety of phenolic compounds have been evaluated as tyrosinase inhibitors or antioxidants but only a few can be active in modulating melanogenesis in melanocytes. Hydroquinone and kojic acid are well-known potent tyrosinase inhibitors but adverse effects limit their use [9]. Ascorbic acid is a widely used antioxidant that is known to inhibit melanogenesis via antioxidant defense but its use is controversial due to its low stability and pro-oxidant activity [10]. These limitations have led to the necessity of developing alternative hypopigmenting agents, for which proteins and peptides from natural sources such as the human body [11,12,13], fungi [14], snails [15], lactic acid bacteria [16] and microalgae [17] or synthetic libraries [18,19,20,21,22] have been investigated as tyrosinase inhibitors. Additionally, food-derived bioactive peptides from honey [23,24], milk [25,26,27], egg white [28] and rice bran [29] have attracted substantial attention because they are small fragments that are isolated from natural proteins, provide beneficial properties and are regarded as safe. Milk protein hydrolysates can prevent oxidative enzymatic browning of food products among which κ-casein and β-lactoglobulin can suppress melanin synthesis in cultured melanocytes by inhibiting tyrosinase activity [25,26,30]. Although milk protein and its hydrolysates are frequently used to inhibit melanin production, no bioactivity of their short peptide fragments has been described previously for hypopigmentation in melanocytes. Bioactive peptides are often in an inactive form within a protein sequence prior to release via a proteolytic process; hence, shorter peptide sequences can have more potent bioactivity. Indeed, many tetrapeptides are pharmaceutically active as minimal fragments and can interact with proteins or receptors affecting a signaling pathway. 

Herein, we aim at defining bioactive tetrapeptide sequences that are derived from milk protein hydrolysates and efficiently downregulate melanin synthesis. We explored the tyrosinase inhibitory activities of peptides that were derived from κ-casein and β-lactoglobulin and were isolated and identified as antioxidants [31,32] since several studies have shown that the antioxidant properties of peptides and their tyrosinase inhibitory activity are related [17,33]. Kim’s group previously reported the dual antioxidant and tyrosinase inhibitory activities of caffeic acid-β-lactoglobulin peptide derivatives [34] but these activities were mainly due to the caffeic acid moiety. Numerous studies have been conducted on the chemical modulation of bioactive compounds via oligopeptide conjugation [35]. However, we focused on the peptide fragments that originate from κ-casein and β-lactoglobulin to provide both antioxidant and tyrosinase inhibitory activity so that hypopigmenting activity in melanocytes would be exhibited by the peptides. We selected four types of milk protein-derived peptides (YFYPEL, YVEEL, MHIRL and WYSLAMAA) that have antioxidant activity and synthesized their peptide fragments that contain four amino acids to identify peptides that exhibited enhanced antioxidant activities, tyrosinase inhibition and eventual hypopigmentation in melanocytes (Table 1).

## 2. Materials and Methods 

### 2.1. Chemicals and Materials

Fmoc-Rink amide linker that was coupled to aminomethyl polystyrene (Rink amide AM) resin (0.82 mmol/g), 4-hydroxymethylfuran-2(5H)-one fritted polypropylene tube reactors (5 mL, Libra tube RT-5 M), benzotriazole-1-yl-oxytris(dimethylamino)phosphonium hexafluorophosphate (BOP), hydroxybenzotriazole (HOBt), Fmoc-*N*-protected amino acids, Ac-MHIR-NH_2_ and MHIR-NH_2_ were purchased from BeadTech (Seoul, Korea). *N*,*N*’-Diisopropylethylamine (DIPEA) was purchased from Alfa Aesar (Haverhill, MA, USA). Ninhydrin, linoleic acid, mushroom tyrosinase and 3,4-dihydroxyphenylalanine (L-DOPA) were purchased from Sigma (St. Louis, MO, USA). Ammonium thiocyanate (NH_4_SCN), ferrous chloride (FeCl_2_) and polyoxyethylenesorbitan monolaurate (Tween 20) were purchased from KANTO Chemicals (Tokyo, Japan). Dimethyl sulfoxide (DMSO), *N*-methyl-2-pyrrolidone (NMP), piperidine, diethyl ether and methanol were obtained from Dae-Jung Chemicals (Siheung-si, Korea). Trifluoroacetic acid (TFA) was purchased from Acros Organics (Fair Lawn, NJ, USA). HPLC gradient acetonitrile was obtained from Future Chem (Seoul, Korea). Dulbecco’s modified Eagle’s medium (DMEM), fetal bovine serum (FBS) and penicillin-streptomycin were purchased from Invitrogen Corporation (Carlsbad, CA, USA). 

### 2.2. Solid Phase Peptide Synthesis 

Milk protein-derived peptides were synthesized on Rink Amide AM resin (0.82 mmol/g) via standard solid-phase peptide synthesis methods with TFA cleavage conditions. The resulting oligopeptides were identified by QUATTRO Triple Quadrupole Tandem mass spectrometer (Micromass & Waters, Milford, MA, USA) at the National Instrumentation Center for Environmental Management (NICEM) and their purities were analyzed by RP-HPLC (Thermo Scientific Spectra System AS300; Thermo-Fisher, Waltham, MA, USA) using a C18 reverse-phase column (120 Å, 5 μm, 4.6 × 250 mm; AAPPTec, Louisville, KY, USA) under the following conditions: gradient elution with A: 0.1% TFA/water, B: 0.1% TFA/acetonitrile; from 10% to 90% over 30 min with a flow rate of 1.0 mL/min; and detection: 230 nm. 

### 2.3. Mushroom Tyrosinase Inhibition Test

A mushroom tyrosinase inhibition test was carried out with L-DOPA as a substrate. Two hundred fifty microliters of phosphate buffer (0.1 M, pH 6.8), 250 µL of 2.5 mM L-DOPA, 200 µL of water and 25 µL of oligopeptide in DMSO at various concentrations were mixed. The reaction mixture was incubated at 25 °C for 10 min after the addition of 25 µL of aqueous mushroom tyrosinase solution (100 µg/mL, 428 U/mL) and then the UV absorbance was measured at 475 nm. The following equation was used to calculate the percentage of mushroom tyrosinase inhibition:

% Tyrosinase Inhibition = (1 − A/B) × 100

where A represents the absorbance at 475 nm of the reaction mixture that contained the inhibitor and B represents the absorbance at 475 nm of a reference reaction mixture that contained DMSO instead of the inhibitor. The concentration of half-maximal inhibitory activity (IC_50_) values were determined by measuring the tyrosinase inhibitory activity at various peptide concentrations (10, 50, 100, 250 and 500 µM). Each experiment was performed in triplicate and the results were averaged. 

### 2.4. Lipid Peroxidation Ferric Thiocyanate Assay

Linoleic acid and Tween 20 were mixed in 0.1 M sodium phosphate buffer (pH 7.0) to prepare a linoleic acid emulsion (50 mM). The linoleic acid emulsion (2.5 mL), 2.0 mL of the phosphate buffer, 0.5 mL of water and 0.5 mL of oligopeptides that were dissolved in methanol were mixed and the final concentration of oligopeptides was 500 µM. These reaction mixtures were tightly capped by septa in glass vials and kept at 50 °C for 30 h under dark conditions. To evaluate the antioxidant activity of the oligopeptides by the quantification of lipid peroxidation inhibition, the ferric thiocyanate assay [36] was used with some modifications. An aliquot of the reaction mixture (25 µL) was withdrawn at intervals and mixed with 1.175 mL of 75% ethanol, 25 µL of 20 mM ferrous chloride in 3.5% HCl and 25 µL of 30% ammonium thiocyanate. The absorbance was measured at 500 nm after 3 min, which was when the color development by the ferric thiocyanate complex was maximal. Each experiment was conducted in triplicate and the results were averaged. 

### 2.5. Cell Culture

B16F10 cells were cultured in DMEM that was supplemented with 10% (v/v) fetal bovine serum (FBS), 50 μg/mL streptomycin and 50 U/mL penicillin at 37 °C in 5% CO_2_. Mel-Ab cells were cultured in DMEM with 10% (v/v) FBS, 100 nM 12-O-tetradecanoylphorbol-13-acetate (TPA), 1 nM cholera toxin (CT), 50 μg/mL streptomycin and 50 U/mL penicillin at 37 °C in 5% CO_2_. 

### 2.6. Cytotoxicity Test

The possible cytotoxicity of the peptide on cells was tested using Cell Counting Kit-8 (CCK-8; CK04, Dojindo, Kumamoto, Japan). The cells were cultured under standard conditions with serum only for the first 24 h and were incubated under starving conditions without serum for the next 48 h to rule out cell growth effects that may affect cell proliferation. Cells were seeded into 96-well plates (B16F10 cells, 5 × 10^3^ per well) or 6-well plates (Mel-Ab cells, 5 × 10^4^ per well). The cultured medium was replaced with serum-free DMEM after 24 h and incubated for another 24 h. Then, the cells were treated with samples (1, 5, 10, 50 and 100 μM) that contained new serum-free media and incubated for 24 h. CCK-8 solution was added according to the manufacturer’s instructions and the cells were incubated for another 2 h at 37 °C. The amount of water-soluble formazan that was generated by the activity of dehydrogenase in cells was measured by the optical density at 450 nm using a SpectraMax Plus Microplate Reader (Molecular Devices, Sunnyvale, CA, USA). All the tests were conducted independently three times. Each experiment was performed in triplicate and the results were averaged.

### 2.7. Melanogenesis Inhibition Test

The effects of the inhibitors (peptides and arbutin) on melanin formation in Mel-Ab cells and B16F10 cells were evaluated. B16F10 cells (5 × 10^3^ per well) were cultured in 96-well plates and incubated with the samples (1, 5, 10, 50 and 100 μM) in a complete medium that contained α-MSH for 4 days. Then, the medium was collected from each well and the optical densities were measured at 405 nm. Mel-Ab cells (5 × 10^4^ per well) were cultured in 6-well plates and incubated with the samples (1, 5, 10, 50 and 100 μM) in the complete medium for 3 days. Next, the treated cells were then dissolved in 1 mL of 1 N NaOH at 100 °C for 30 min and centrifuged for 20 min at 16,000 g, after which the optical densities of the supernatants were measured at 400 nm. 

The percentage of melanin content was calculated via following equation:

% melanin content = (A/B) × 100

where A represents the optical density of the sample-treated group and B represents the optical density of the untreated control group. All the tests were conducted independently three times. Each experiment was performed in triplicate and the results were averaged.

For the Western blot analysis, B16F10 cells were seeded into 6-well plates. After 24 h, the cultured medium was replaced with serum-free DMEM and incubated for another 24 h. Then, the cells were treated with samples in new serum-free media and incubated for 0–360 min. Cells were lysed with RIPA buffer containing protease inhibitor cocktail and phosphatase inhibitor cocktail. Protein concentration was determined using the Bicinchoninic acid (BCA) protein assay kit (Thermo Scientific, Rockford, IL, USA). Protein (20 μg) samples were separated on 4–20% polyacrylamide gels and then transferred to polyvinylidene fluoride (PVDF) membranes (#1704156, Bio-Rad, Hercules, CA, USA), which were blocked with 5% nonfat dry milk in Tris buffered saline solution containing 0.1% Tween 20. The blots were incubated with the primary antibodies at 4 °C overnight. Membrane-bound primary antibodies were detected using HRP-conjugated secondary antibodies and chemiluminescent substrate. Digital images of the blotted membranes were obtained and analyzed using ChemiDoc XRS+ Systems with Image Lab Software (Version 5.1) (Bio-Rad). The relative protein expression of p-cAMP response element-binding protein (CREB) was normalized to that of CREB and glyceraldehyde 3-phosphate dehydrogenase (GAPDH).

## 3. Results and Discussion

### 3.1. Synthesis of Peptides

We synthesize ĸ-casein or β-lactoglobulin-derived peptides (YFYPEL, YVEEL, MHIRL and WYSLAMAA), their tetrapeptide derivatives (YFYP, FYPE, YPEL, YVEEL, VELL, YVEL, MHIR, HIRL, WYSL, YSLA, SLA, LAMA and AMAA) and modified HIRL peptides (FIRL, AIRL, HILL, HIEL and HIKL) to evaluate the hypopigmenting activity (Table 1). The peptides were synthesized at high purity (approximately 90% or higher) (Table S1) and their tyrosinase inhibitory activities and antioxidant activities were measured as the purity of each peptide was considered to ensure consistency of the concentrations used. Their biological activities were evaluated in melanocytes and tyrosinase kinetic analysis after further purification using a semipreparative RP-HPLC column. The peptides were identified via mass spectrometry: MHIRL (m/z calcd: 668.4 [M + H]+; found: 668.5), MHIR (m/z calcd: 555.7 [M + H]+; found: 555.3), HIRL (m/z calcd: 537.7 [M + H]+; found: 537.4), WYSLAMAA (m/z calcd: 911.4 [M + H]+; found: 911.3), WYSL (m/z calcd: 567.7 [M + H]+; found: 567.2), YSLA (m/z calcd: 452.5 [M + H]+; found: 452.2), SLAM (m/z calcd: 420.5 [M + H]+; found: 420.1), LAMA (m/z calcd: 403.5 [M + H]+; found: 404.1), AMAA (m/z calcd: 362.5 [M + H]+; found: 362.1), YFYPEL (m/z calcd: 830.4 [M + H]+; found: 830.3), YFYP (m/z calcd: 588.7 [M + H]+; found: 588.3), FYPE (m/z calcd: 554.6 [M + H]+; found: 554.2), YPEL (m/z calcd: 519.6 [M + H]+; found: 520.3), YVEEL (m/z calcd: 651.3 [M + H]+; found: 651.3), YVEE (m/z calcd: 538.6 [M + H]+; found: 538.1), VEEL (m/z calcd: 488.6 [M + H]+; found: 488.2), YVEL (m/z calcd: 522.6 [M + H]+; found: 522.2), FIRL (m/z calcd: 588.7 [M + H]+; found: 588.3), AIRL (m/z calcd: 554.6 [M + H]+; found: 554.2), HILL (m/z calcd: 519.6 [M + H]+; found: 520.3), HIEL (m/z calcd: 651.3 [M + H]+; found: 651.3) and HIKL (m/z calcd: 538.56 [M + H]+; found: 538.1).

### 3.2. Mushroom Tyrosinase Inhibition Activity

**Figure 1:**
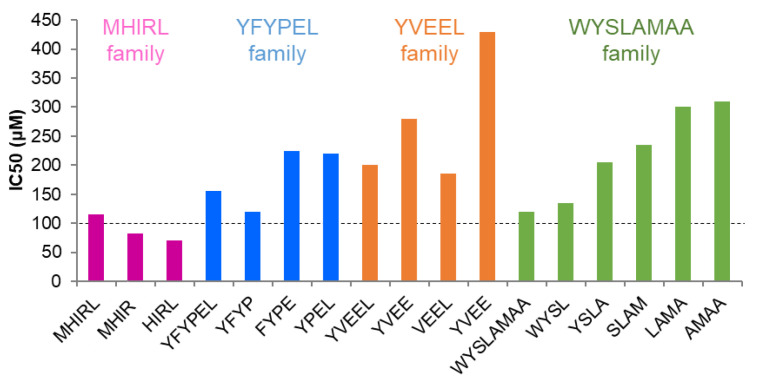
Half-maximal inhibitory concentration (IC_50_) values of milk-derived peptides and their fragments. The dotted line indicates tetrapeptides under IC_50_ 100 µM. Conditions: various concentrations of peptides, L-DOPA (2.5 mM) and mushroom tyrosinase (100 µg/mL, 428 U/mL) were incubated at 25 °C for 10 min.

The concentration of half-maximal inhibitory activity (IC_50_) of synthesized peptides was investigated for the comparison of their tyrosinase inhibitory activities (Figure 1). Methionine-histidine-isoleucine-arginine amide (MHIR) and histidine-isoleucine-arginine-leucine amide (HIRL) showed low IC_50_ values of 83 μM and 70 μM, respectively (Figure S1); hence, they are potent tyrosinase inhibitory peptides. Although the sulfur atom of methionine (M) in MHIR and methionine-histidine-isoleucine-arginine-leucine amide (MHIRL) may interact with the copper ions in the active site of tyrosinase [37], the presence of methionine appears to be insignificant for the inhibition of tyrosinase because the IC_50_ value of HIRL is lower than that of MHIR. 

**Figure 2:**
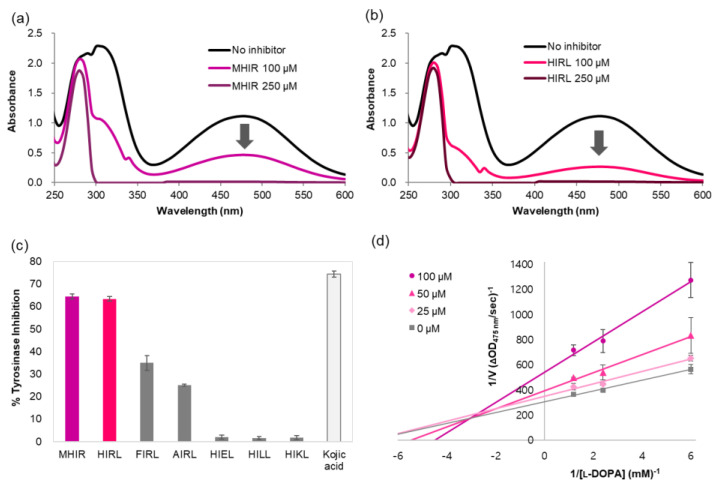
The effects of peptides (**a**) methionine-histidine-isoleucine-arginine amide (MHIR) and (**b**) histidine-isoleucine-arginine-leucine amide (HIRL) on dopachrome formation with L-DOPA and mushroom tyrosinase, (**c**) the mushroom tyrosinase inhibitory activities of MHIR, HIRL derivatives and kojic acid. The percent tyrosinase inhibition was determined after treating 100 μM of peptides or kojic acid with L-DOPA (2.5 mM) and mushroom tyrosinase (100 µg/mL, 428 U/mL) and incubating at 25 °C for 10 min. Each experiment was conducted in triplicate and the results were averaged. (**d**) Lineweaver-Burk plot of tyrosinase inhibition reaction in the presence of MHIR peptide at various concentrations.

By UV-Vis scanning, we show that MHIR and HIRL can reduce melanin production. MHIR and HIRL sufficiently suppress dopachrome formation at 475 nm and let the substrate L-DOPA remain in the reduced form at 282 nm (Figure 2a,b). We also synthesized a series of modified HIRL peptides, which showed the lowest IC_50_ values and used them to understand the tyrosine inhibition mechanism and to determine whether each amino acid in HIRL affects the tyrosinase inhibitory activity of HIRL (Figure 2c). Kojic acid was used as a positive control. When histidine was replaced by phenylalanine (**F**IRL) or aliphatic alanine (**A**IRL), the tyrosinase inhibitory activity was reduced to 53% or 38%, respectively, compared to that of HIRL. When arginine was replaced by negatively charged glutamic acid (HI**E**L), aliphatic leucine (HI**L**L) or positively charged amino acid lysine (HI**K**L), the tetrapeptides almost completely lost their tyrosinase inhibitory activity. These results demonstrate that the aromatic ring at the first amino acid position and arginine at the third amino acid position plays a vital role in the tyrosinase inhibitory activity of HIRL. The Lineweaver-Burk plot exhibits the tyrosinase inhibition mode of MHIR peptide. With increasing concentrations of MHIR peptide, the K_m_ values increase and the V_max_ values decrease that indicates a mixed-mode of inhibition (Figure 2d). 

Other peptides that we tested had moderate tyrosinase inhibitory activity, with IC_50_ values of 115–430 μM (Figure 1). In the YFYPEL family (YFYP, FYPE and YPEL), YFYP showed higher tyrosinase inhibitory activity than YFYPEL, while FYPE and YPEL exhibited significantly lower tyrosinase inhibitory activity than YFYPEL. In the YVEEL family (YVEE, VEEL and YVEL), VEEL had slightly lower IC_50_ value than YVEEL but YVEE and YVEL had 1.4–2.2 times higher IC_50_ values than YVEEL. The presence of tyrosine (Y) at the *N*-terminus does not appear to be essential as previously reported [38]. In the WYSLAMAA family (WYSL, YSLA, SLAM, LAMA and AMAA), only WYSL showed similar tyrosinase inhibitory activity to WYSLAMAA. The inhibitory activity of other tetrapeptides in the WYSLAMAA family sharply decreased by 1.7–2.6 times compared to the activity of the original peptide.

### 3.3. Antioxidant Activity

**Figure 3:**
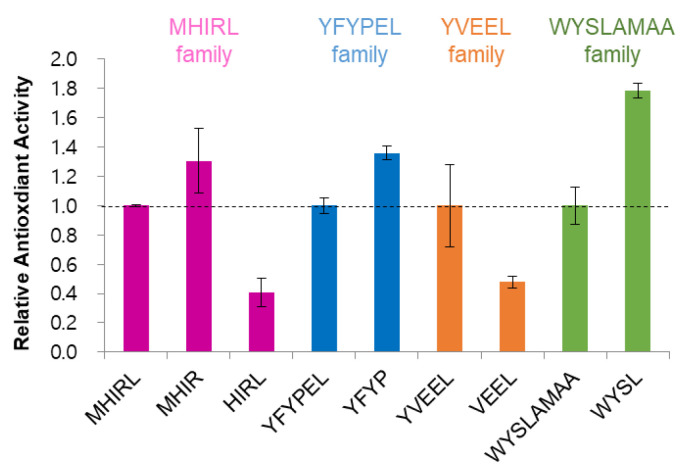
Relative antioxidant activities of peptides that are based on the percentage of lipid peroxidation inhibition after 24 h. The reaction was conducted at 50 °C under dark conditions. The final concentration of peptides was 250 μM. Each experiment was performed in triplicate and the values are presented as the mean ± standard error.

To identify the tetrapeptides that exhibit a dual antioxidant and tyrosinase inhibition activity, tetrapeptides that exhibit excellent tyrosinase inhibition activity are selected from each peptide family and these peptides are evaluated in terms of antioxidant activity. A lipid substrate (linoleic acid) formed a peroxide (R-O-O-R’) by oxidative degradation reaction and therefore, lipid peroxide quantification is used for evaluating antioxidant potential [39], which can be monitored by ferric thiocyanate complexation (Figure S2). To facilitate comparison, we set the relative inhibition of lipid peroxidation of each original oligopeptide to 1.0 and calculated the relative antioxidant activity of the tetrapeptide fragments (Figure 3). MHIR, YFYP and WYSL showed enhanced antioxidant activity by 1.3–1.8 times compared to the original peptides (MHIRL, YFYPEL and WYSLAMAA). In contrast, the antioxidant activity of HIRL and VEEL decreased by more than 50%, which may have been due to the lack of antioxidant amino acid residues such as methionine or tyrosine.

### 3.4. Melanogenesis Inhibition in Melanocytes

**Figure 4:**
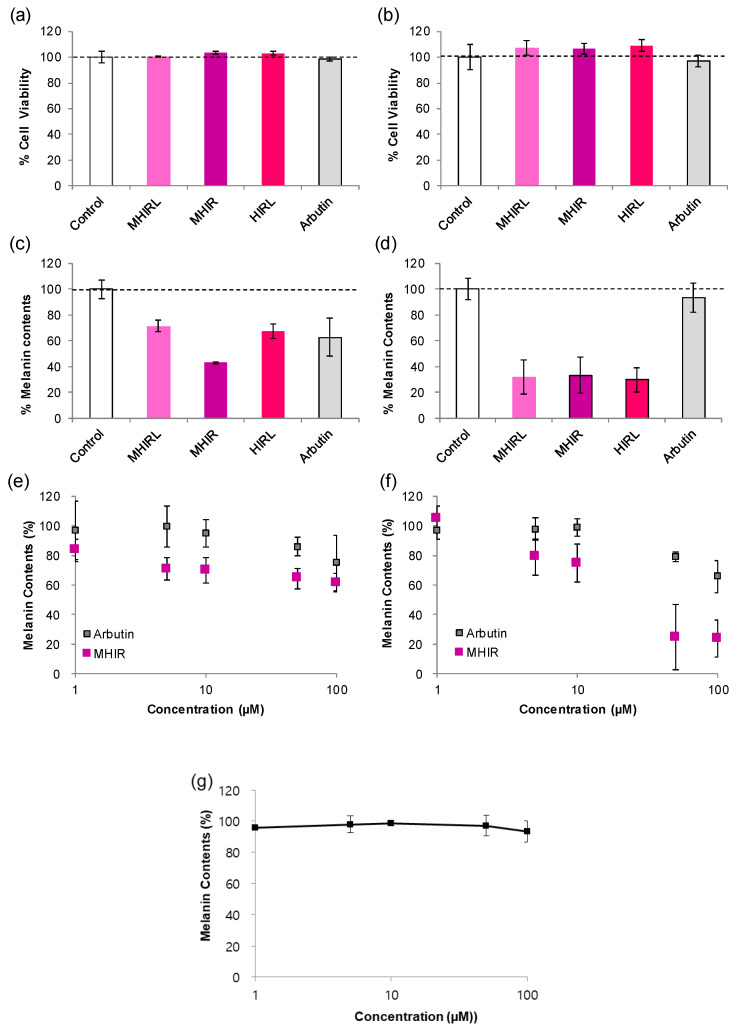
Hypopigmenting activity of methionine-histidine-isoleucine-arginine-leucine (MHIRL) family peptides in melanocytes. The effects of MHIRL family on cell viability (**a**) in Mel-Ab cells and (**b**) in B16F10 cells. [peptide] = 100 μM. The melanogenesis inhibitory activities of the MHIRL family (**c**) in Mel-Ab cells and (**d**) in B16F10 cells. [peptide] = 100 μM. The melanogenesis inhibitory activities of MHIR (**e**) in Mel-Ab cells and (**f**) in B16F10 cells. (**g**) Remaining melanin contents (%) in α-MSH treated B16F10 cells with *N*-terminal acetylated MHIR (Ac-MHIR) at various concentrations. Each experiment was carried out in triplicate and the values are presented as the mean ± standard error.

We selected MHIRL family peptides that showed excellent tyrosinase inhibitory activity and antioxidant activity and determined whether these peptides could reduce melanin production in melanocytes. Typically, the results depend on the cell type. Mel-Ab is a mouse-derived spontaneously immortalized melanocyte cell line that produces copious amounts of melanin in culture [40]. Mel-Ab requires the continual presence of an activator of protein kinase C, such as 12-O-tetradecanoylphorbol-13-acetate (TPA) for cell proliferation but comparable treatment with B16 results in partial inhibition of growth [41]. The administration of α-MSH strikingly induces melanogenesis of the B16F10 melanoma cell line through an increase in tyrosinase activity. However, Mel-Ab produces a large amount of melanin in the culture medium without α-MSH. The selected peptides showed minimal cytotoxicity at 100 µM in both types of melanocytes, Mel-Ab cells and B16F10 cells (Figure 4a,b). The melanogenesis inhibitory activity of the MHIRL family in melanocytes is evaluated in comparison with that of arbutin, which is widely used as a hypopigmenting agent in the industry. In Mel-Ab cells, MHIR reduced the melanin content by 57% at 100 µM, thereby demonstrating higher activity than arbutin and MHIRL and HIRL moderately decreased the melanin content by 28 and 33%, respectively (Figure 4c). This can explain the combined effect of MHIR on hypopigmentation with potent tyrosinase inhibitory activity and antioxidant activity. Interestingly, all MHIRL family peptides decreased melanin production by 70% in B16F10 cells that were stimulated by α-MSH (Figure 4d). When we examined the melanin contents (%) at various MHIR concentrations, MHIR reduced the melanin content by 30% at 5 μM and by up to 40% at higher concentrations in Mel-Ab cells (Figure 4e). MHIR suppressed the melanin production by up to 80% at 50 µM in B16F10 cells (Figure 4d).

From this result, we hypothesize that MHIR may possess antagonistic properties toward the type 1 melanocortin receptor (MC1R) by competing with α-MSH and attenuating the melanin-producing signaling pathway. The core peptide of α-MSH is known as HFRW (α-MSH6-9). The negatively charged regions of MC1R (Glu94, Asp117 and Asp121) interact with positively charged arginine (R) and aromatic amino acids, such as phenylalanine (F) and tryptophan (W) interact with the TM4, TM5 and TM6 regions [42]. Based on the interaction of α-MSH with MC1R and our knowledge of the amino acids in α-MSH, we can produce peptide sequences that are involved in the melanogenesis process. Tetrapeptide FRWG was reported to be the minimal inhibitory sequence of α-MSH [8]. Recently, tetrapeptide RFWG and RLWG, along with tripeptide RWG, showed anti-melanogenesis activity that was comparable to that of FRWG in B16F10 cells [18]. In our study, MHIRL, MHIR and HIRL, which consist of a positively charged aromatic imidazole and a positively charged aliphatic chain, have structural similarities with the inhibitory sequence of α-MSH and may interfere with melanin synthesis. Interestingly, we also identified an important role of the *N*-terminus. When the *N*-terminus of MHIR is acetylated (Ac-MHIR), it barely inhibits melanin production in α-MSH treated B16F10 cells (Figure 4g).

**Figure 5:**
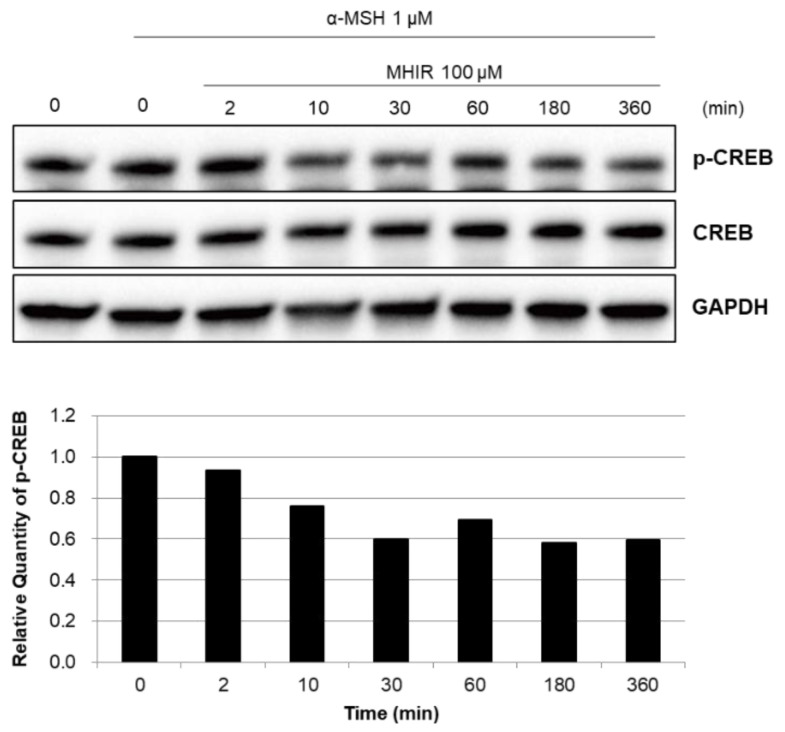
Effects of the MHIR peptide on melanogenesis-related signaling. α-MSH-treated B16F10 cells were incubated with MHIR (100 μM) for 0~360 min, after which the whole-cell lysates were analyzed via western blotting using specific antibodies. Equal protein loading was confirmed by probing for GAPDH expression. The relative quantities of phosphorylated CREB were normalized to that of total CREB. This graph is a representative result of three independent experiments.

To demonstrate that MHIR interferes with α-MSH-induced melanin production, we treated B16F10 cells with 100 µM of MHIR up to 360 min (Figure S3). It is clearly observed that MHIR suppresses phosphorylation of the cyclic adenosine 5′-monophosphate response element binding protein (CREB) at serin-133 (Figure 5) by 18% in 10 min and by 37% in 180 min. Since α-MSH binding to MC1R results in phosphorylation of CREB (p-CREB), this low p-CREB possibly indicated that MHIR could interfere with α-MSH binding to MC1R [43].

## 4. Conclusions

We synthesized ĸ-casein (YFYPEL) and β-lactoglobulin-derived antioxidant peptides (MHIRL, YVEEL and WYSLAMAA) and their tetrapeptide derivatives to investigate whether they can be utilized for skin hypopigmentation. β-Lactoglobulin-derived tetrapeptides, MHIR and HIRL, showed potent tyrosinase inhibitory activities with the IC_50_ values of 83 µM and 70 µM, respectively. These tyrosinase inhibition activities are comparable to that of kojic acid, the most intensively studied tyrosinase inhibitor with IC_50_ values of 10–300 µM [44]. We found that aromatic amino acids and arginine played essential roles in inhibiting tyrosinase. When the histidine or arginine of HIRL was replaced with an aliphatic amino acid, the peptide lost its tyrosinase inhibitory activity. YFYP, which is a tetrapeptide that is derived from ĸ-casein, along with MHIR, HIRL and WYSL, which are tetrapeptides that are derived from β-lactoglobulin, showed satisfactory tyrosinase inhibitory activity and improved antioxidant activity by 1.3–1.8 times compared to the original peptides. We tested the peptides against mushroom tyrosinase and thus, inhibition profile may be slightly different from human tyrosinase [4]. Melanogenesis inhibition tests have proven that MHIRL, MHIR and HIRL, which exhibit excellent tyrosinase inhibitory activity and antioxidant activity, reduced melanin production in melanocytes through a complex process of inhibiting oxidation and tyrosinase reaction. Especially, MHIR can interfere with α-MSH binding to MC1R in B16F10 cells, consequently lower CREB phosphorylation, and, finally, attenuate melanogenesis. Despite the differences in mechanisms and efficiency, MHIR can reduce the melanin contents of both Mel-Ab and B16F10 cells. Our study shows that MHIR is the active tetrapeptide that is obtained from natural protein hydrolysates and can effectively act as a potential hypopigmenting agent in both Mel-Ab and B16F10 cell systems. Peptides are generally considered safe and the synthesis of tetrapeptides can be scalable and cost-effective. Therefore, we believe that this peptide, which is originated from milk-protein hydrolysates, can be widely used in the fields of cosmetics, pharmaceuticals, food and agriculture.

## Figures and Tables

**Table 1:** Milk protein-derived peptides and their tetrapeptide fragments.

Source	Original Peptide Sequence	Tetrapeptide Fragments		
κ-casein	YFYPEL	YFYP	FYPE	YPEL
β-lactoglobulin	YVEEL	YVEE	VEEL	YVEL
	MHIRL	MHIR	HIRL	
	WYSLAMAA	WYSL	YSLA	SLAM
		LAMA	AMAA	

Note. Y = tyrosine; F = phenylalanine; P = proline; E = glutamic acid; L = leucine; V = valine; M = methionine; H = histidine; I = isoleucine; R = arginine; W = tryptophan; S = serine; A = alanine.

## Supplementary Materials

The following are available online at https://www.mdpi.com/2076-3921/9/11/1106/s1, Table S1: Purity of peptides, Figure S1: Determination of IC_50_ values of MHIRL peptide family, Figure S2: Lipid peroxidation inhibition (%) of antioxidant peptides, Figure S3: Effects of the MHIR peptide on melanogenesis-related signaling.
