# Supplementary material for: Milk Protein-Derived Antioxidant Tetrapeptides as Potential Hypopigmenting Agents
Source: Antioxidants (Basel). 2020 Nov 10;9(11):1106. doi: 10.3390/antiox9111106 (PMC7698045; doi:10.3390/antiox9111106)
Supplement: Supplementary file 1 [file antioxidants-09-01106-s001.pdf]

Supplementary Materials:

Milk Protein-derived Antioxidant Tetrapeptides as Potential Hypopigmenting Agents

Saerom Kong[1,†], Hye-Ryung Choi[2,3,†], Yoon-Jeong Kim[2], Yoon-Sik Lee[1,*], Kyoung-Chan Park[2,4,*] and Seon-Yeong Kwak[5,6,*]

Table S1. Purity of peptides

Peptide	HPLC Purity (%)	Peptide	HPLC Purity (%)
YFYPEL family		MHIRL family	
YFYPEL	90	MHIRL	89
YFYP	>99	MHIR	95
FYPE	>99	HIRL	93
YPEL	>99	WYSLAMAA family	
YVEEL family		WYSLAMAA	90
YVEEL	90	WYSL	91
YVEE	>99	YSLA	98
VEEL	90	SLAM	87
YVEL	>99	LAMA	97
		AMAA	92

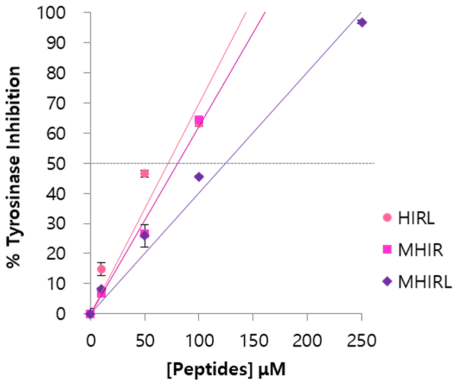

Figure S1. Determination of IC_{50} values of MHIRL peptide family.

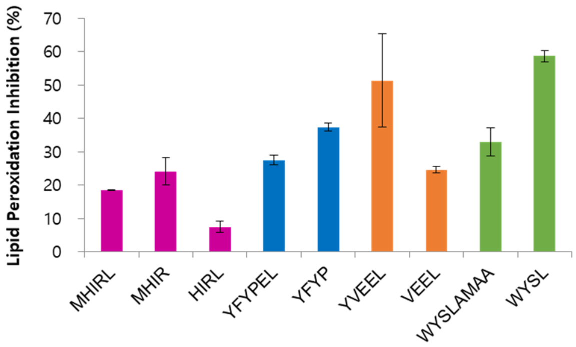

Figure S2. Lipid peroxidation inhibition (%) of antioxidant peptides.

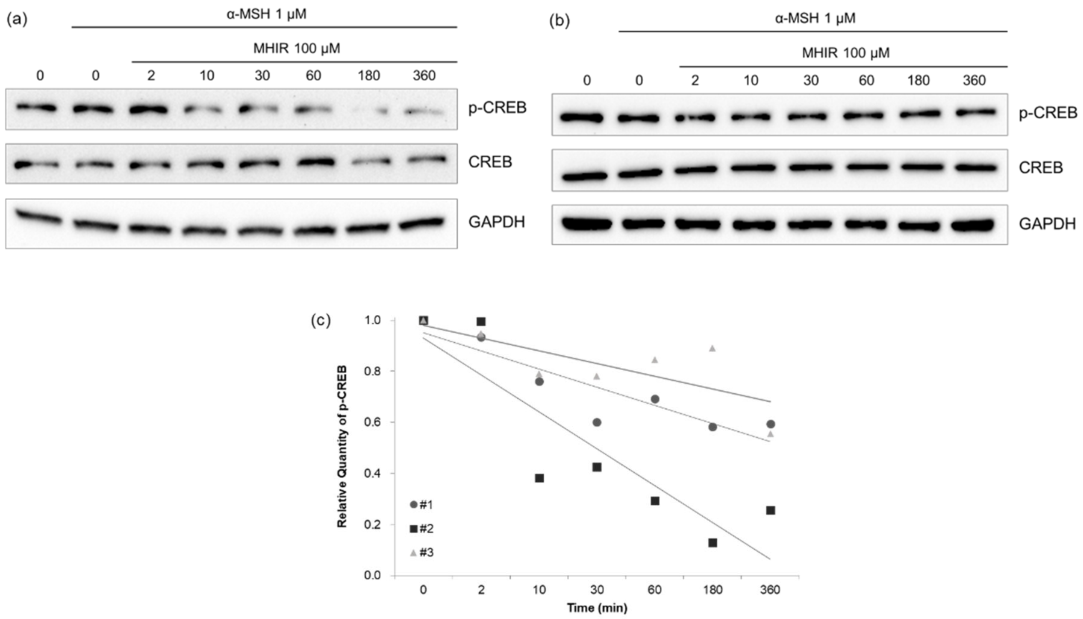

Figure S3. Effects of the MHIR peptide on melanogenesis-related signaling. The graph (a) and (b) show data from three independent experiments. The CREB phosphorylation was reduced in a time-dependent manner, but was not statistically significant. The graph (c) show all data from three independent experiments (The legend means the each experimental set 1, 2, and 3).
